# Construction of a hypoxia-immune-related prognostic panel based on integrated single-cell and bulk RNA sequencing analyses in gastric cancer

**DOI:** 10.3389/fimmu.2023.1140328

**Published:** 2023-04-26

**Authors:** Cuncan Deng, Guofei Deng, Hongwu Chu, Songyao Chen, Xiancong Chen, Xing Li, Yulong He, Chunhui Sun, Changhua Zhang

**Affiliations:** ^1^ Digestive Diseases Center, The Seventh Affiliated Hospital, Sun Yat-sen University, Shenzhen, China; ^2^ Guangdong Provincial Key Laboratory of Digestive Cancer Research, The Seventh Affiliated Hospital of Sun Yat-sen University, Shenzhen, Guangdong, China

**Keywords:** gastric cancer, hypoxia, prognostic panel, immune infiltration, immune therapy

## Abstract

**Introduction:**

Gastric cancer (GC) is the fifth most common tumor, contributing to the third-highest number of cancer-related deaths. Hypoxia is a major feature of the tumor microenvironment. This study aimed to explore the influence of hypoxia in GC and establish a hypoxia-related prognostic panel.

**Methods:**

The GC scRNA-seq data and bulk RNA-seq data were downloaded from the GEO and TCGA databases, respectively. AddModuleScore() and AUCell() were used to calculate module scores and fractions of enrichment for hypoxia-related gene expression in single cells. Least absolute shrinkage and selection operator cox (LASSO-COX) regression analysis was utilized to build a prognostic panel, and hub RNAs were validated by qPCR. The CIBERSORT algorithm was adopted to evaluate immune infiltration. The finding of immune infiltration was validated by a dual immunohistochemistry staining. The TIDE score, TIS score and ESTIMATE were used to evaluate the immunotherapy predictive efficacy.

**Results:**

Hypoxia-related scores were the highest in fibroblasts, and 166 differentially expressed genes were identified. Five hypoxia-related genes were incorporated into the hypoxia-related prognostic panel. 4 hypoxia-related genes (including POSTN, BMP4, MXRA5 and LBH) were significantly upregulated in clinical GC samples compared with the normal group, while APOD expression decreased in GC samples. Similar results were found between cancer-associated fibroblasts (CAFs) and normal fibroblasts (NFs). A high hypoxia score was associated with advanced grade, TNM stage, N stage, and poorer prognosis. Decreased antitumor immune cells and increased cancer-promoting immune cells were found in patients with high hypoxia scores. Dual immunohistochemistry staining showed high expression of CD8 and ACTA2 in gastric cancer tissue. In addition, the high hypoxia score group possessed higher TIDE scores, indicating poor immunotherapy benefit. A high hypoxia score was also firmly related to sensitivity to chemotherapeutic drugs.

**Discussion:**

This hypoxia-related prognostic panel may be effective in predicting the clinical prognosis, immune infiltrations, immunotherapy, and chemotherapy in GC.

## 1 Introduction

Gastric cancer (GC) is the fifth most prevalent cancer globally, causing the third most cancer-related death worldwide (1). Exceeding 1 million individuals have been diagnosed with GC, and 784000 deaths were caused by GC worldwide in 2018. Although some advances have been achieved in both diagnosis and therapy, the survival rate of GC is still unsatisfactory in many countries (2). The current dilemma of gastric cancer includes the lack of effective early diagnosis, poor clinical outcomes, and high metastasis and recurrence rates.

The tumor microenvironment consists of inflammatory cells, cancer-associated fibroblasts (CAFs), nerves, and vascular endothelial cells (3). The interaction of components in the tumor microenvironment promotes tumour progression. Hypoxia is a vital feature of the tumor microenvironment (TME) in solid tumors and is associated with various cancer features, such as metabolic reprogramming, impaired immune response, and increased genomic instability (4). Hypoxia can enhance tumor cell proliferation, immune escape, and inflammation, induce angiogenesis and activate invasion, consequently leading to the aggression, metastasis, and drug resistance of gastric cancer (5, 6). Hypoxia is associated with tumor malignancy progression, treatment resistance, and poor clinical prognostic outcomes for patients (7). Hypoxia-related genes improve proliferation and distant metastasis through the miR-30c-2-3p/LOX axis in GC (8). The downregulation of miR-4521 caused by hypoxia inhibits the progression of gastric carcinoma by regulating the expression of IGF2 and FOXM1 (9). The lncRNA-CBSLR, which is induced by hypoxia, regulates ferroptosis in gastric cancer by modulating CBS through a m6A-YTHDF2-dependent mechanism (10). SERPINE1 and EFNA3 might be hypoxia-related prognostic factors in GC (11). Hypoxia-induced lncRNAs could facilitate the invasion of GC by interacting with SNAI1 (12).

At present, several studies have elaborated on the mechanism by which hypoxia regulates the physiological changes in gastric cancer, but the mechanism needs further elucidation. Elucidating the hypoxia-related pathogenesis and identifying effective biomarkers of gastric cancer are meaningful for improving the diagnosis, prevention and management of GC.

In this study, we aimed to develop a hypoxia-related prognostic panel to predict the immune microenvironment (TME) in GC patients. First, GC scRNA-seq data were obtained from the GEO database, and bulk RNA-seq data were obtained from the TCGA database. The hypoxia hallmark genes were utilized to calculate the hypoxia score and AUC value. Least absolute shrinkage and selection operator Cox (LASSO-COX) regression analysis was utilized to build a novel hypoxia score-related prognostic panel. The CIBERSORT algorithm was manipulated to analyze the relationship between the infiltration of immune cells and the hypoxia score. The tumor immune dysfunction and exclusion (TIDE) score and T-cell-inflamed signature (TIS) score were used to evaluate the immunotherapy predictive efficacy of the hypoxia score.

## 2 Methods

### 2.1 Data acquisition

**Figure 1:**
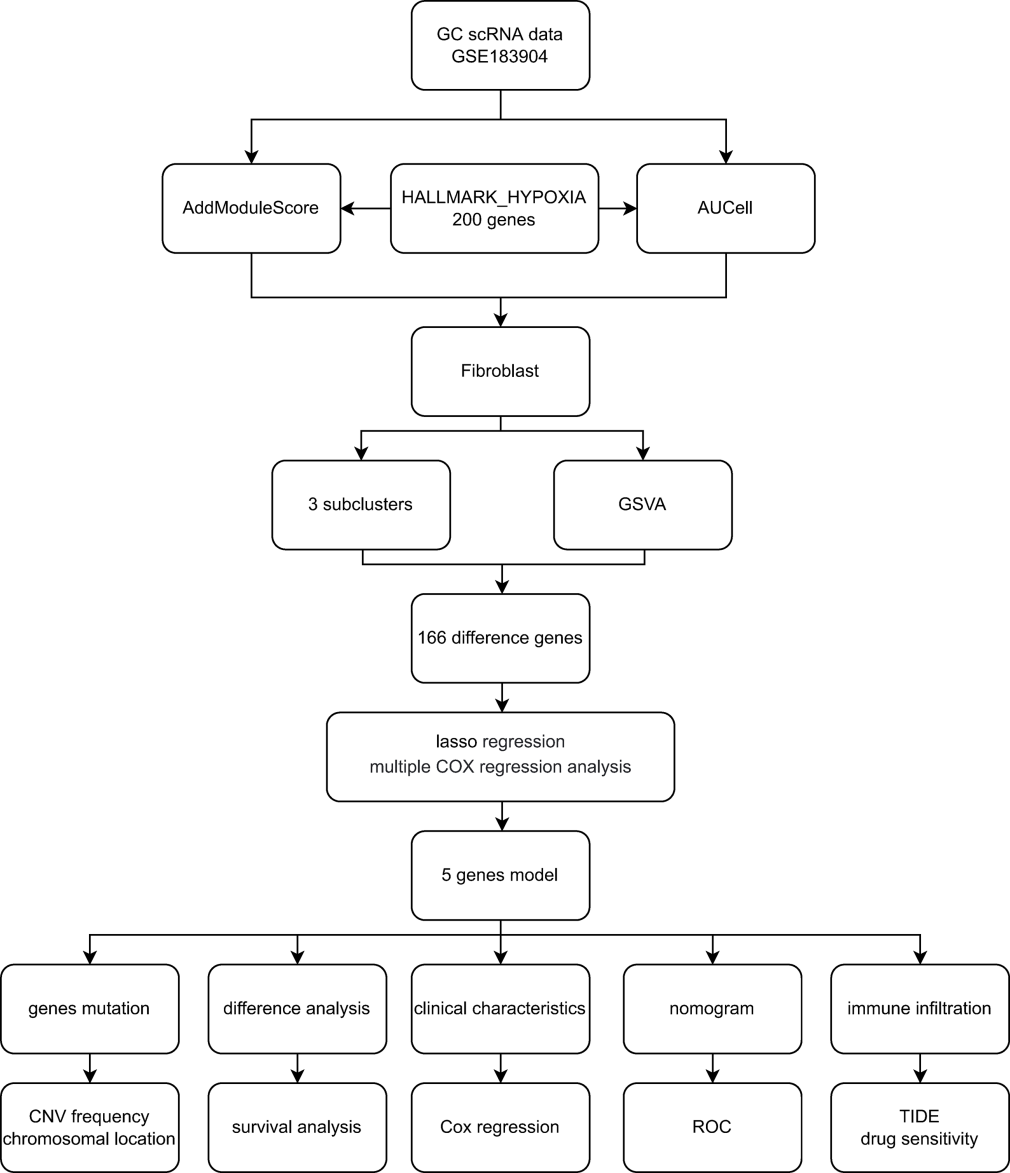
Flowchart of the study.

The processing flow of this research is shown in **Figure 1**. The GC scRNA-seq data GSE183904 were accessed from the GEO database (https://www.ncbi.nlm.nih.gov/geo/), which included 10 normal tissue samples, 26 GC tissue samples, 3 peritoneum tissue samples from GC patients and 1 normal peritoneum tissue sample. Bulk RNA-seq data for GC were accessed from the TCGA database (https://portal.gdc.cancer.gov/) comprising 32 normal tissues and 375 gastric cancer tissues. Clinical data and survival data were also retrieved.

### 2.2 Processing of scRNA-seq data

GC scRNA-seq data were analyzed by using the single-cell analysis R package “Seurat”. The preliminary data screening process was carried out according to this standard: the number of genes detected in a single cell was more than 200 and less than 5,000, and the mitochondrial gene count was 20%.

The SCTransform() function of the Seurat package was utilized to preprocess and reduce the batch effect to integrate different single-cell transcriptome samples, and 5000 highly variable genes were chosen by SelectIntegrationFeatures() for anchoring. Then, RunPCA() was adopted to reduce the dimension of PCA with dim = 20 to further reduce dimensionality with the UMAP method according to ElbowPlot(), and the resolution was set to 0.4 for cluster analysis using the FindClusters() function. Uniform Manifold Approximation and Projection (UMAP) is an algorithm that reduces dimensionality by mapping a high-dimensional probability distribution to a low-dimensional space. Finally, we identified 10 cell types based on typical cell markers.

### 2.3 Score according to hypoxia-related hallmark genes

To calculate module scores and the fraction of enrichment for hypoxia-related gene expression in single cells, AddModuleScore() and AUCell() were performed. The hypoxia hallmark gene was downloaded from the Molecular Signatures Database (http://www.gsea-msigdb.org/gsea/msigdb/index.jsp), which incorporates 200 genes widely employed in cancer research. We calculated the hypoxia score and AUC value in each cell type with the 200 hypoxia-related hallmark genes.

### 2.4 Gene set variation analysis

GSVA was used to sequence the different genes in normal and GC tissue, aiming at evaluating the enrichment of hallmark gene sets in the differential gene list. DEGs were screened using FindMarkers(), with the expression ratio of the least differential genes set to 0.25. The hallmark gene sets were accessed from the MSigDB database with the R package “msigdbr”, species = “Homo sapiens”, category = “H”.

### 2.5 Least absolute shrinkage and selection operator cox regression analysis

To formulate the hypoxia-based prognostic panel, LASS analysis was employed to select reliable genes related to the clinical outcome of GC patients from the TCGA cohort. We calculated the hypoxia score for every GC patient with the following formula: score= Σ coefficient mRNAn * expression level mRNA. Patients were divided into two groups according to the calculated scores. Follow-up analysis will focus on patients with different hypoxia scores.

### 2.6 Clinical specimen collection and ethics approval

Gastric cancer and normal samples were accessed from the Seventh Hospital of Sun Yat-sen University. The sample collection procedure was approved by the Sun Yat-sen University Health Science Institution Review Board (No. KY-2022-051-02). All tissues were preserved at -80 degrees for further study.

### 2.7 Cell isolation and cell culture

The GC cell line MKN45 and normal control GES1 cells were purchased from Shanghai Institute of Cell Biology, Chinese Academy of Sciences (Shanghai, China). MKN45 and GES1 cells were cultured using RPMI 1640 medium (Gibco) containing 10% fetal bovine serum (FBS, Nanjing BioChannel Biotechnology Co., Ltd., China) in a 37°C, 5% CO2 environment.

To isolate cancer-associated fibroblasts and normal fibroblasts (NFs), gastric cancer tissues and normal tissues were obtained from the Seventh Hospital of Sun Yat-sen University respectively. The tissues were washed three times with PBS containing 1% penicillin streptomycin. The tissue was cut into 1-3 mm pieces using surgical scissors and then digested by adding collagenase IV and shaking for 1-2 hours at 37°C. The precipitate was obtained by centrifugation at 1000 rpm for 10 minutes and the red blood cells were then lysed by adding erythrocyte lysis solution(C3702, Beyotime, China). Digestion was terminated by the addition of a high sugar Dulbecco’s Modified Eagle Medium (11965092, DMEM, Gibco) containing 10% serum and 1% penicillin and streptomycin. The obtained cells are cultured in a CO2 incubator at 37°C with 5% CO2.

### 2.8 Real-time PCR analysis of hub RNAs identified by LASSO

Several hub RNAs were identified by LASSO analysis. The expression of these hub RNAs was detected by qPCR. Total RNA was extracted from the gastric tissues and cell lines (including GES1 cells, MKN45 cells, NFs and CAFs) with the AG RNAex Pro RNA reagent (Accurate Biology, CAT#AG21102) following the manufacturer’s instructions. cDNA was synthesized using Evo M-MLV reverse transcription master mix (Accurate Biology, CAT# AG11706). qPCR was conducted utilizing a SYBR Green Pro Tag HS premixed qPCR kit (Accurate Biology, CAT# AG11701). The relative expression of the hub RNAs was calculated using the 2^–ΔΔCt^ method. mRNA expression was normalized to β-actin. The primer sequences of all RNAs used for qPCR are recorded in **Table S1**.

### 2.9 Hypoxia-related gene analysis

The “findMarkers” function within the Seurat package was utilized to investigate the expression levels of five genes across distinct cell types in single-cell sequencing data. Kaplan-Meier analysis of selected hypoxia-related genes in the TCGA-GC cohort. GEPIA2 database (http://gepia2.cancer-pku.cn/) to explored the associations between CAFs markers (ACTA2, FAP) and genes included in the prognostic panel in gastric cancer.

### 2.10 Univariate and multivariate Cox regression analysis

To verify whether the hypoxia score was an independent prognostic factor, we performed a Cox regression analysis. The variables included in the univariate Cox regression analysis included age, sex, tumor grade, TNM stage and hypoxia score, and significant factors were included in the multivariate Cox regression analysis. The results are shown in a forest diagram.

### 2.11 Clinical correlation and survival analysis

For a deeper understanding of the relationship between the hypoxia score and clinical features, clinical correlation analysis was conducted among patients in different groups. Furthermore, we utilized Kaplan−Meier (K-M) analysis to find differences in OS outcomes between the high- and low-score groups. A time-dependent receiver operating characteristic (ROC) curve was generated to determine the predictive ability of the risk model.

### 2.12 Immune cell infiltration

To analyze the relationship between the infiltration of immune cells and the hypoxia score, the CIBERSORT (HTTPS://cibersort.stanford.edu/) (13) algorithm was adopted to evaluate the infiltration of immune cells in TCGA-GC patients. The Wilcoxon test was applied to analyze the difference in infiltrated immune cells between the high- and low-score groups. The infiltration difference of some functional cells in different score groups was also evaluated with the same method.

### 2.13 Dual immunohistochemistry staining

The finding of immune infiltration was validated by a dual immunohistochemistry staining. A dual immunohistochemistry staining kit (#DS-0003, ZSGB-BIO, China) was used following the manufacturer’s protocols to assess the association of CD8+ T cell and CAFs in GC tissues. The sections of GC tissue, which had been fixed in formalin and embedded in paraffin, were subjected to deparaffinization in xylene 20 minutes after being heated in an oven at 65°C for 2 hours. Following this, they were rehydrated in 100%, 95%, 85%, and 75% alcohol for 2 minutes each. Antigen retrieval was performed with Citrate solution. All slides were blocked with goat serum buffer at 37°C for 30 min and then incubated with CAFs marker Anti-ACTA2 (1:100, Genxspan, #GXP6460) and CD8 (1:100, Huabio, # ET1606-31) primary antibodies at 4°C overnight. The next day, the slides were incubated with AP-labeled Rabbit and HRP-labeled mouse secondary antibodies at 37°C for 1 hours. Then, the related products were detected with DAB and RED respectively. The nuclei were stained for 1 to 2 minutes using hematoxylin. Finally, the sections were dehydrated, transparent and sealed with gum. The slides were viewed with a microscope and images captured.

### 2.14 Immunotherapy prediction

To predict the prognostic value of hypoxia scores in immunotherapy patients, time-dependent receiver operating characteristic (ROC) curve analysis was adopted to acquire the area under the curve (AUC). In addition, the tumor immune dysfunction and exclusion (TIDE) score and T-cell-inflamed signature (TIS) score were downloaded online (HTTP://tide.dfci.harvard.edu/) to compare the prognosis among the hypoxia scores, TIDE, and TIS by multiple ROC curves.

### 2.15 Analysis of the purity of tumors using ESTIMATE

The Estimation of Stromal and Immune cells in Malignant Tumours using Expression data (ESTIMATE) algorithm was employed to calculate the scores of stromal cells, immune cells and tumor cells in the different hypoxia score groups (14). The contents of immune cells and stromal cells in the tumor microenvironment (TME) were obtained for further analysis of the relationship between the hypoxia score and the purity of the tumor.

### 2.16 Drug sensitivity analysis

The sensitivity of different drugs was predicted in GC patients in the high-hypoxia score subgroup and low-hypoxia score subgroup. The R package pRRophetic was employed to predict drug sensitivity. Significant differences in IC50 between the high and low hypoxia score subgroups were evaluated with the Wilcoxon signed-rank test. The result was visualized with the package “ggplot2”.

### 2.17 Statistical analysis

R software (version 4.1.2; https://www.R-project.org) and associated R packages were applied in data management, such as the “limma” package for difference analysis between different groups and the “Survminer” package for survival analysis. The Wilcoxon test was conducted to compare the differences among distinct groups. The Spearman correlation method was conducted to calculate the correlation coefficient. All statistical analyses were bilateral, P<0.05. 0.05 was considered statistically significant.

## 3 Result

### 3.1 Annotation of cell types and hypoxia score

**Figure 2:**
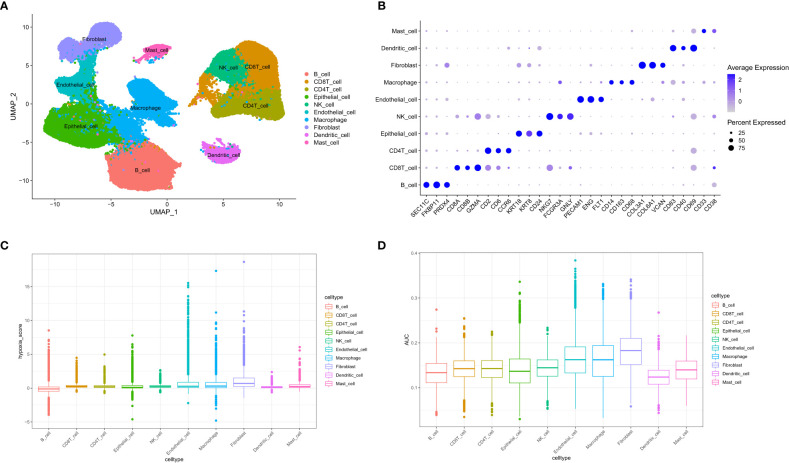
Overview of single-cell data. **(A)** UMAP of ten different cell types of samples. **(B)** Cell markers used to identify cell types. **(C)** Hypoxia score calculated by the AddModuleScore function. **(D)** Hypoxia-related AUC calculated by the AUCell function.

**Figure 3:**
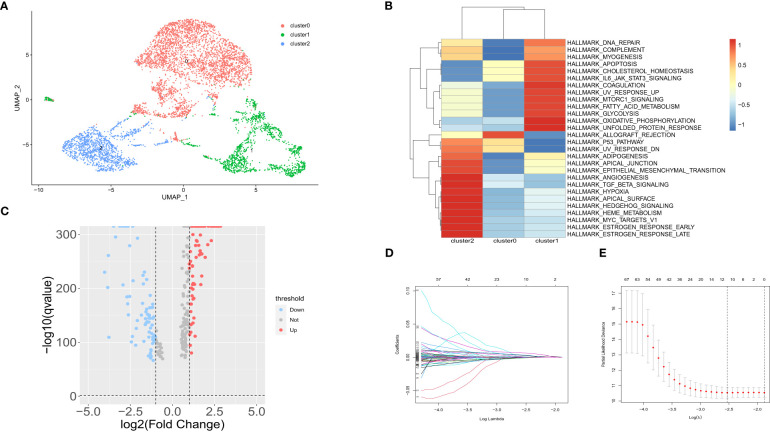
Subpopulation analysis in fibroblast subtypes. **(A)** UMAP of 3 fibroblast clusters. **(B)** Hallmarks enriched by GSVA in 3 fibroblast clusters. **(C)** Volcano maps of up- and downregulated genes in fibroblast Cluster 2. **(D)** LASSO coefficient distribution of each independent gene. **(E)** The partial likelihood deviance in LASSO Cox regression analysis.

A total of 73981 cells and 26571 genes were screened from GSE183904. Ten cell types were annotated according to typical cell markers (**Figure 2A**). The cell markers for annotation are shown in **Figure 2B**. Hypoxia-related scores and AUCs were the highest in fibroblasts compared with other cell types based on the AddmoduleScore function (**Figure 2C**) and AUCell (**Figure 2D**). Therefore, fibroblasts were extracted for subsequent analysis. Three clusters were obtained by secondary clustering of fibroblasts (**Figure 3A**). Then, the GSVA enrichment score was determined for each cell in the fibroblast subcluster, and the results indicated that the hypoxia-related hallmark was enriched in Cluster 2 of fibroblasts (**Figure 3B**).

### 3.2 Differential gene analysis

The DEGs between fibroblast Cluster 2 and other clusters were screened by the FindMarkers function (logFC>1, p value<0.05, Minpct = 0.25). 166 differentially expressed genes in cluster 2 CAFs between normal and gastric cancer group were identified that were used for follow-up analysis. The volcano map of the differentially expressed genes is shown in **Figure 3C**. LASSO-Cox regression analysis was conducted to identify the hub genes. The change trajectory of genes is shown in **Figures 3D, E**. Finally, 5 genes were screened as hypoxia-related genes to construct the hypoxia-related prognostic model, including APOD, BMP4, POSTN, MXRA5 and LBH.

### 3.3 qPCR validation and Kaplan−Meier analysis of genes included in LASSO model

**Figure 4:**
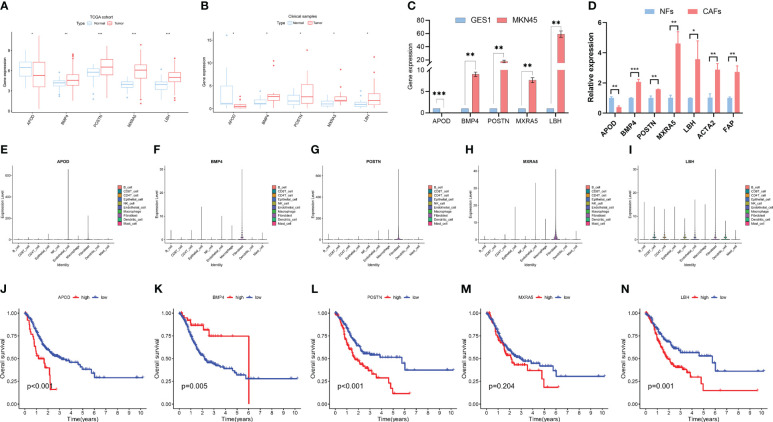
Characteristics of 5 hypoxia-related genes in gastric cancer. **(A)** Expression differences of 5 hypoxia-related genes in normal and gastric cancer tissues in the TCGA dataset. **(B)** Validation of the expression of 5 hub genes by PCR in clinical patients. **(C)** Validation of the expression of 5 hub genes by PCR in cell lines. **(D)** Validation of the expression of 5 hub genes by PCR in NFs and CAFs. **(E–I)**. The expressions of 5 hub genes in different cells according to scRNA-seq data. **(J–N)**. Kaplan−Meier analysis of patients in high- and low- expression groups of these 5 hub genes. * means p<0.05; ** means p<0.01; *** means p<0.001.

The differentially expressed genes in the LASSO model were analyzed. Clinical gastric cancer samples were collected to perform qPCR. In terms of expression level, 4 hypoxia-related genes (including POSTN, BMP4, MXRA5 and LBH) were significantly upregulated in clinical GC samples compared with the normal group, while APOD expression decreased in GC samples (TCGA cohort-**Figure 4A**; clinical samples-**Figure 4B**). Furthermore, the expression of these hub genes was detected by PCR in cell lines. Consistent with the tissue results, POSTN, BMP4, MXRA5 and LBH were significantly upregulated and APOD was decreased in MKN45 cells compared with normal control cells (**Figure 4C**). Besides, we detected the expressions of genes included in the prognostic panel in NFs and CAFs. The CAFs marker ACTA2 and FAP significantly upregulated in CAFs compared with NFs. POSTN, BMP4, MXRA5 and LBH were significantly upregulated and APOD was decreased in CAFs compared with NFs (**Figure 4D**).

We further explored the expressions of these 5 genes in the scRNA-seq data. The results showed that these genes were highly expressed in fibroblast but also expressed in other cells, including Endothelial cell, epithelial cell, B cell, CD4 T cell and CD8 T cell et al. (**Figures 4E–I**).

Furthermore, Kaplan−Meier (K-M) analysis was performed to explore the correlation between RNA expression and survival in GC. The result showed that high APOD, POSTN, MXRA5 and LBH expression was related to a poor prognosis in GC while high BMP4 expression was associated with a higher survival rate (**Figures 4J–N**).

There were many differentially expressed genes in cluster 2 CAFs compared with other cluster CAFs and the genes were listed in **Table S2**. Since ACTA2 and FAP were the well-known markers of CAFs, the correlation between CAFs markers (ACTA2 and FAP) and hypoxia-related prognostic panel (including APOD, POSTN, BMP4, MXRA5 and LBH) were analysed using GEPIA2 database. The results were showed in **Figure S1**.

### 3.4 Clinicopathologic characteristics analysis and model construction

**Figure 5:**
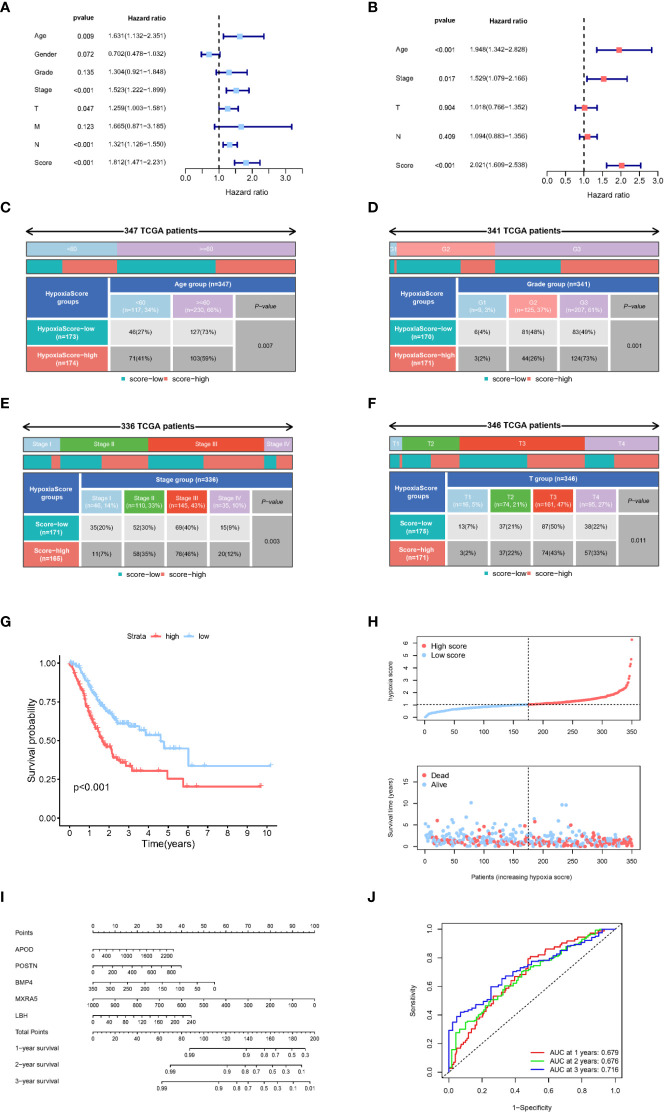
Clinical characteristics of the hypoxia-related gene model. **(A)** Univariate Cox regression analysis of clinical characteristics and hypoxia scores. **(B)** Multivariate Cox regression analysis of significant clinical characteristics and hypoxia scores. **(C–F)**. Age, grade, tumour stage and T stage were significantly different between the two hypoxia score subgroups. **(G)** Kaplan−Meier analysis of patients in different hypoxia score groups. **(H)** Relationship between survival status and hypoxia score in TCGA-GC patients. **(I)** The nomogram constructed with 5 hypoxia-related genes to predict the 1-, 3-, and 5-year OS in GC patients in the TCGA cohort. **(J)** ROC curves of key hypoxia genes for predicting 1-, 3-, and 5-year OS in the TCGA cohort.

According to the results of LASSO-Cox regression analysis, the hypoxia score was calculated using gene expression and coefficients. TCGA-GC patients were divided into high and low groups according to the median hypoxia score. According to univariate Cox regression analysis, TNM stage, T stage, N stage and hypoxia scores were significantly related to the prognosis of gastric cancer (**Figure 5A**). In further exploration, **Figure 5B** shows that the hypoxia score was an independent prognostic factor in multivariate Cox regression analysis. The clinicopathologic characteristics of GC patients in the TCGA cohort showed a significant difference in age, TNM grade, TNM stage and T stage between the high and low hypoxia score groups (**Figures 5C–F**, **S2A**). There was no significant difference in N stage, M stage or gender (**Figures S2B-D**). KM survival analysis showed a significantly poorer prognosis in the higher hypoxia score group than in the low hypoxia score group (**Figure 5G**). The results of survival analysis for each candidate gene are shown in **Figure S3**. **Figure 5H** shows the relationship between the hypoxia score and patient survival status, and higher scores suggest a worse prognosis. Finally, we used the selected hypoxia-related genes to build a prognostic correlation prediction nomogram (**Figure 5I**). As the ROC curve shows, the hypoxia-related gene model could effectively predict the prognosis of GC patients, and the area under the curve (AUC) value reached 0.679 at 1 year, 0.676 at 2 years, and 0.716 at 3 years (**Figure 5J**).

### 3.5 Association between immune infiltration and hypoxia score

**Figure 6:**
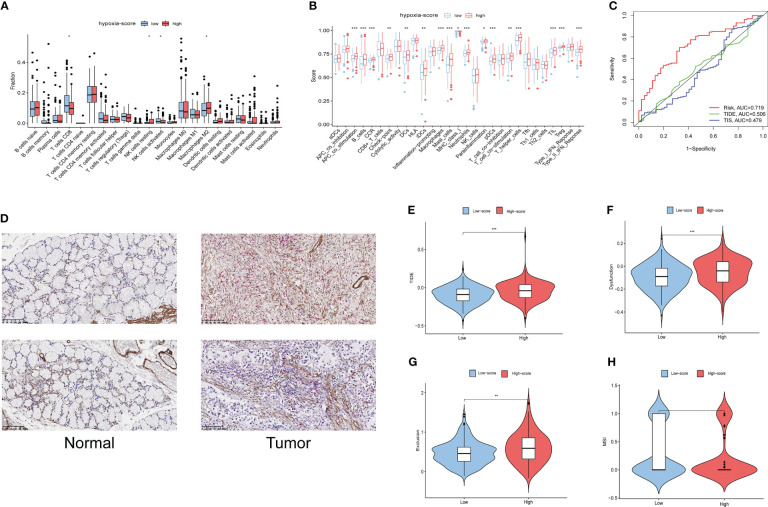
Immune infiltration and prognostic values of immunotherapy in different hypoxia score groups. **(A)** The fractions of 22 immune cells between the low and high hypoxia score groups by the CIBERSORT method. **(B)** The scores of 29 functional cells between the low and high hypoxia score groups. **(C)** ROC curves of the hypoxia score, TIDE and TIS to predict the OS of GC patients in TCGA cohorts. **(D)** The finding of immune infiltration was validated by a dual immunohistochemistry staining(ACTA2 and CD8). **(E–H)**. Differences in TIDE, T-cell exclusion score, T-cell dysfunction score and MSI in the two hypoxia score subgroups. * means p<0.05; ** means p<0.01; *** means p<0.001.

CIBERSORT was used to estimate the infiltration of 22 immune cells in the TCGA-GC cohort, and then the difference in immune cell infiltration in the different hypoxia score groups was explored. The results showed that antitumour immune cells (including activated NK cells or CD8+ T cells) were fewer in the high hypoxia score patients, while cancer-promoting immune cells such as resting NK cells and M2 macrophages were increased in the high score group (**Figures 6A, B**). The majority of functional immune cells infiltrated the high hypoxia score group, indicating that the hypoxia score was closely related to the immune microenvironment. The correlation analysis between hypoxia genes and immune cells is shown in **Figure S4A**, which indicated that M2 macrophages and activated B cells were positively correlated with the hypoxia score, while neutrophils and activated memory CD4 T cells were the opposite (**Figures S4B-4E**).

This LASSO model was built based on the DEGs in cluster 2 CAFs. To validate the relationship of CAFs and immune infiltration, we used double-staining immunohistochemistry to detect the CAF marker ACTA2 and the CD8+ T cell marker CD8.The result showed that the expression of ACTA2 was upregulated in the gastric cancer accompanied with the high expression of CD8. This result showed that CAFs is associated with immune infiltration (**Figure 6D**).

### 3.6 Immunotherapy predictive efficacy of the hypoxia score

The Tumour Immune Dysfunction and Exclusion (TIDE) algorithm was adopted to test the interactions between candidate genes and cytotoxic T-cell function. The TIDE predictive score is positively related to immune evasion, proving resistance to immunotherapy. According to the ROC curve, the hypoxia score is a better prognostic panel than the TIDE score or the TIS score (**Figure 6C**). In the TCGA-GC cohort, the TIDE score of the high hypoxia score group was significantly higher than that of the low score group (**Figure 6E**). Furthermore, the T-cell exclusion scores (**Figure 6F**) and the T-cell dysfunction score (**Figure 6G**) were significantly different between the two hypoxia score subgroups, except for the MSI score (**Figure 6H**). These results indicated that patients with a high hypoxia score show poor immunotherapy benefit, which is consistent with the findings of previous studies. Survival analysis suggests that patients with a high hypoxia score have a poor prognosis (15).

**Figure 7:**
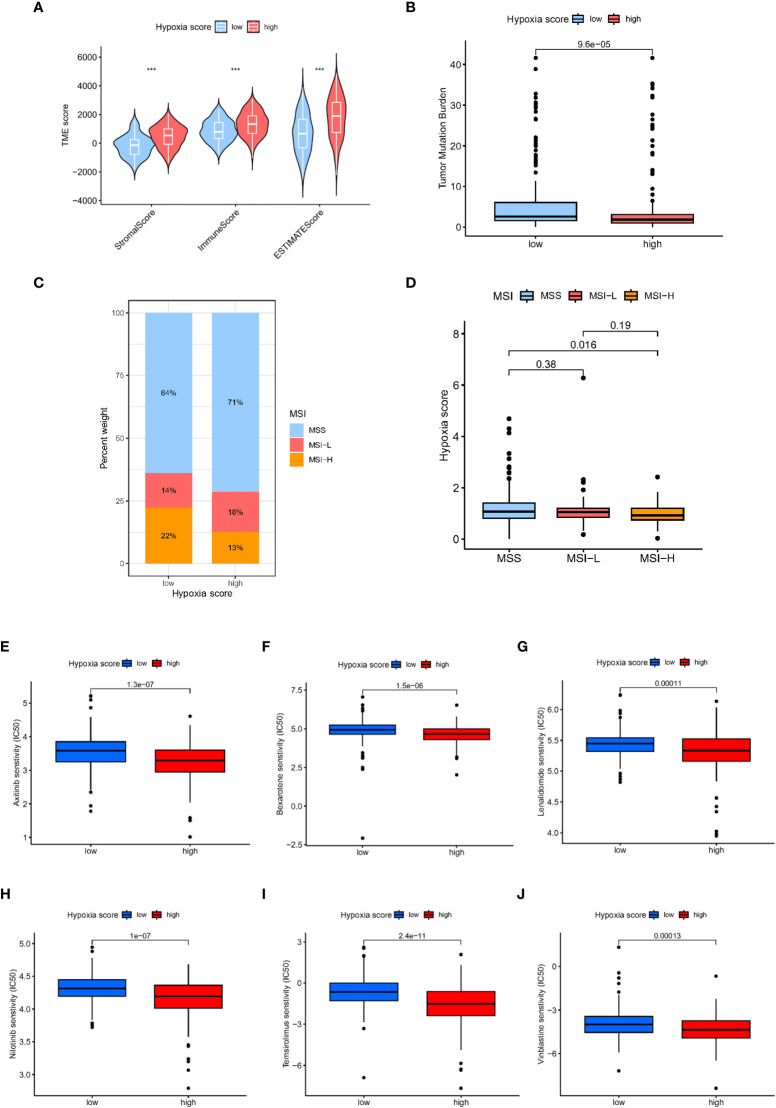
Analysis of tumour microenvironment and drug sensitivity in patients with different hypoxia scores. **(A)** TME scores in patients with different hypoxia scores based on ESTIMATE. **(B)** The TMB in different hypoxia score groups. **(C, D)** Relationship between hypoxia score and MSI. **(E)** Relationship between hypoxia score and tumour stemness index. **(E–J)**: Sensitivity analysis of hypoxia scores and antineoplastic drugs.

According to the ESTIMAT algorithm, the patients with high hypoxia scores also had higher tumour purity than the patients with low hypoxia scores (**Figure 7A**). Tumour mutational burden (TMB) was defined as the total number of somatic mutations detected in every one million bases. Studies have shown that patients with a high tumour mutational burden are more likely to benefit from ICI treatment. The expression of TMB was remarkably upregulated in the low hypoxia score group compared with the high hypoxia score group (**Figure 7B**). Moreover, we observed that a low hypoxia score was associated with MSI-H status, while a high CAFS score was associated with microsatellite stable (MSS) status (**Figures 7C, D**).

### 3.7 Drug sensitivity

Furthermore, we explored the relationship between the hypoxia score and the effectiveness of chemotherapy for GC treatment. We discovered that a high hypoxia score was associated with a lower half inhibitory concentration (IC50) of chemotherapeutics, including axitinib, bexarotene, lenalidomide, nilotinib, temsirolimus and vinblastine (**Figures 7E–J**, P<0.05). Therefore, our study indicated that the hypoxia score could serve as a potential effective predictor of chemotherapy sensitivity prediction.

## 4 Discussion

Gastric cancer, the third major cause of cancer-related deaths worldwide, exhibits a worse clinical prognosis and elevated metastasis rate. The hypoxic TME is present in almost all solid tumors and profoundly affects the progression of gastric cancer (10). A hypoxic tumor microenvironment is one of the characteristics of gastric cancer. Gastric cancer cells in the microenvironment can influence the biological properties of tumor cells by affecting the expression of certain transcription factors and tumor-associated genes to adapt to the hypoxic environment. Tumours are usually tolerant to anticancer drugs under hypoxic conditions. Although hypoxia has been reported to participate in proliferation, aggression, metastasis and drug resistance, the deeper mechanisms remain to be elucidated.

In our study, 10 cell types were identified from GC scRNA-seq data, and hypoxia-related scores were the highest in fibroblasts. The tumor microenvironment contains miscellaneous cells, including fibroblasts, immune cells, nerves, and vascular endothelial cells, which can interact with cancer cells (16). Cancer-associated fibroblasts (CAFs) are one of the most abundant constituents of the cancer microenvironment. Tumour-associated fibroblasts interact with tumor cells and other stromal components, such as immune cells, to promote gastric cancer progression. Activated CAFs can produce chemokines, extracellular matrix, growth factors and metabolites. These molecules can interact with tumor cells and facilitate tumor growth and inflammatory responses through direct contact or in a paracrine manner. Tumour cells switch their metabolic state between oxidative phosphorylation and glycolysis by establishing metabolic interactions with CAFs. In the hypoxic environment, tumor cells exhibit the Warburg effect, but in the normoxic environment, they exhibit the reverse Warburg effect. Metabolic interactions between CAFs and tumor cells facilitate the proliferation and metastasis of gastric cancer (17). CAFs usually play a tumor-promoting role, but recent studies have found that CAFs may also have an inhibitory effect on tumor progression (18, 19).

To further clarify the mechanism of hypoxia in gastric cancer, we screened differentially expressed genes in fibroblasts and used LASSO-Cox analysis to construct a novel hypoxia-related prognostic panel. As a result, 5 hypoxia-related genes (including APOD, POSTN, BMP4, MXRA5, and LBH) were included in the prognostic model. In agreement with our results, genes involved in this prognostic panel were significant in hypoxia-related physiological processes. APOD is a potential biomarker of hypoxia and is involved in immune responses (20). APOD is included in a novel necrosis-related gene model for predicting the prognosis of gastric adenocarcinoma and is closely associated with the immune microenvironment of cold tumors (21). In glioma, POSTN may regulate resistance to anti-VEGF-A therapy by upregulating the expression of TGFβ1 and HIF1α (22). POSTN is implicated in promoting metastasis of ovarian cancer via its ability to enhance M2 macrophages and cancer-associated fibroblasts through integrin-mediated activation of the NF-κB and TGF-β2 signaling pathways (23). Zhong et al. reported that BMP4 may play an important role in regulating glycolysis in hepatocellular carcinoma cells under hypoxia and hypoglycemia (24). BMP4 Promotes Tumor Progression in Bladder Cancer by Inducing M2 Macrophage Polarization (25). Hypoxia could accelerate malignant progression in glioma by promoting the expression of LBH (26). LBH inhibits cellular migration, invasion and epithelial-mesenchymal transition in nasopharyngeal carcinoma via downregulating αB-crystallin expression (27). MXRA5 was involved in a 6-gene prognostic stratification system which can be used to evaluate the prognostic risk (28). These genes involved in our prognostic panel are closely associated with hypoxia or immune cells and could promote tumor progression.

According to this five-gene prognostic panel constructed by LASSO-Cox regression analysis, TCGA-GC patients were classified into high and low hypoxia score groups. We found a remarkable difference in grade, TNM stage and N stage between the high and low hypoxia score groups. Patients with higher hypoxia scores have a significantly poorer prognosis than individuals with low hypoxia scores. The ROC curve shows that the hypoxia-related prognostic panel could effectively predict the prognosis of GC patients. These results indicated that hypoxia is a poor prognostic factor for gastric cancer. Consistent with a previous study, studies showed that the hypoxia-induced factor HIF-1α could facilitate the migration, proliferation, invasion, and tumor angiogenesis of gastric cancer cells (29). Hypoxia is involved in GC cell proliferation, migration and invasion through activation and upregulation of NHE1 (30). Hypoxia influences the expression of a variety of genes (including HIF-1α and von Hippel Lindau protein (pVHL)), resulting in the progression of cancer (31, 32).

Hypoxia is an essential feature of the TME [6], while immune infiltration is another prominent feature (33, 34). In our study, we found that antitumor immune cells, including CD8+ T cells and activated NK cells, were less abundant in the high hypoxia score patients, while cancer-promoting immune cells, such as resting NK cells and M2 macrophages, were more abundant in the high score group. The TIDE score, T-cell dysfunction scores and exclusion score of the high hypoxia score group were significantly increased compared with those of the hypoxia score subgroups. These results suggest that patients with a high hypoxia score show poor immunotherapy benefit, which is consistent with the results of a previous study. A signature of genes related to both hypoxia and immune response has been developed for the purpose of predicting the risk stratification and survival outcomes in individuals with triple-negative breast cancer (35). Hypoxia significantly upregulates PD-L1 expression in immune cells in a HIF-1α-dependent manner (36). Hypoxia promotes the activity of immunosuppressive cells and immune escape, mediating adaptation to the hypoxic environment in cancer cells (37, 38). Hypoxia is firmly associated with an immunosuppressive microenvironment and can promote gastric cancer progression.

In addition to the TIDE score, the IPS can also reflect the expression level of immune checkpoints, which can reflect the response sensitivity to ICI treatment. An effective model of patient selection based on hypoxia prior to ICI treatment of gastric cancer has not been established. Our study indicated that the expression of immune checkpoints (ICs) is closely related to the hypoxia score, which was remarkably higher in the low hypoxia score group. Patients in the low-score group are more likely to stimulate an immune response and were sensitive to immunotherapy. Consistent with our results, previous studies proved that hypoxia inhibited immune surveillance by regulating the expression of immune checkpoints comprising CTLA-4, PD-1 or PD-L1 (39). Hypoxia is an obstacle to tumor immunotherapy (40). This hypoxia-related prognostic model may be meaningful for guiding clinical immunotherapy.

Furthermore, we wondered whether the usage of a combination of chemotherapy and immunotherapy in GC had better efficacy. Therefore, we explored the chemotherapy sensitivity of various agents in the high and low hypoxia score subgroups of gastric cancer patients. Our study discovered that the high hypoxia score group had a high potential for ICI response to chemotherapeutics, including axitinib, bexarotene, lenalidomide, nilotinib, temsirolimus and vinblastine. According to the hypoxia score, these drugs predicted possible potential for therapeutic drugs under certain conditions (41, 42).

Our study innovatively combines single-cell sequencing data with a hypoxia gene set to build a novel prognostic model for gastric cancer. We innovatively found that the hypoxia-related gene set was enriched in cluster 2 cancer-associated fibroblasts (CAFs), and constructed a novel prognostic model by using the differential genes in this group of cells through LASSO algorithm. There were several limitations in our study. First, some data lack clinical follow-up information, which needs further experimental research and a larger sample size for verification. In addition, a direct clinical application test of the prognostic model is needed. We will further verify the predictive ability of this prognostic model through clinical samples in subsequent studies.

## 5 Conclusion

A novel five-element hypoxia-related panel established based on single-cell and bulk RNA sequencing is a potential biomarker for gastric cancer prognostic prediction. This hypoxia-related prognostic panel was firmly associated with immune infiltration, immunotherapy and chemotherapy. This study may provide potential targets for GC therapy, but more experimental research is needed.

## Data availability statement

The datasets presented in this study can be found in online repositories. The names of the repository/repositories and accession number(s) can be found within the article/**Supplementary Materials**.

## Ethics statement

The studies involving human participants were reviewed and approved by Sun Yat-sen University Health Science Institution Review Board. The patients/participants provided their written informed consent to participate in this study.

## Author contributions

Conceptualization: CD and CZ; data curation, methodology, and software: CD, GD, and HC; supervision and funding acquisition: YH, CS, and CZ; project administration and validation: SC, XC, and XL; writing-original draft: CD and GD; writing – review and editing: CS and CZ. All authors contributed to the article and approved the submitted version.
