# Supplementary material for: Construction of a hypoxia-immune-related prognostic panel based on integrated single-cell and bulk RNA sequencing analyses in gastric cancer
Source: Front Immunol. 2023 Apr 26;14:1140328. doi: 10.3389/fimmu.2023.1140328 (PMC10169567; doi:10.3389/fimmu.2023.1140328)
Supplement: Supplementary file 1 [file DataSheet_1.docx]

Supplementary Material

Construction of a hypoxia-immune-related prognostic panel based on integrated single-cell and bulk RNA sequencing analyses in gastric cancer

Cuncan Deng^1†^, Guofei Deng^1†^, Hongwu Chu^1†^, Songyao Chen^1^, Xiancong Chen^1^, Xing Li^1^, Yulong He^1,2^, Chunhui Sun^1,2*^, Changhua Zhang^1,2*^.

^1^Digestive Diseases Center, The Seventh Affiliated Hospital, Sun Yat-sen University, Shenzhen, China.

^2^Guangdong Provincial Key Laboratory of Digestive Cancer Research, The Seventh Affiliated Hospital of Sun Yat-sen University, Shenzhen, Guangdong, People’s Republic of China.

^†^These authors contributed equally to this work.

*** Correspondence:**Chunhui sun, [sunchh6@mail.sysu.edu.cn](mailto:sunchh6@mail.sysu.edu.cn); Changhua Zhang, [zhchangh@mail.sysu.edu.cn](mailto:zhchangh@mail.sysu.edu.cn).

# Supplementary Figures and Tables

For more information on Supplementary Material and for details on the different file types accepted, please see [here](https://www.frontiersin.org/guidelines/author-guidelines#supplementary-material).

## Supplementary Figures


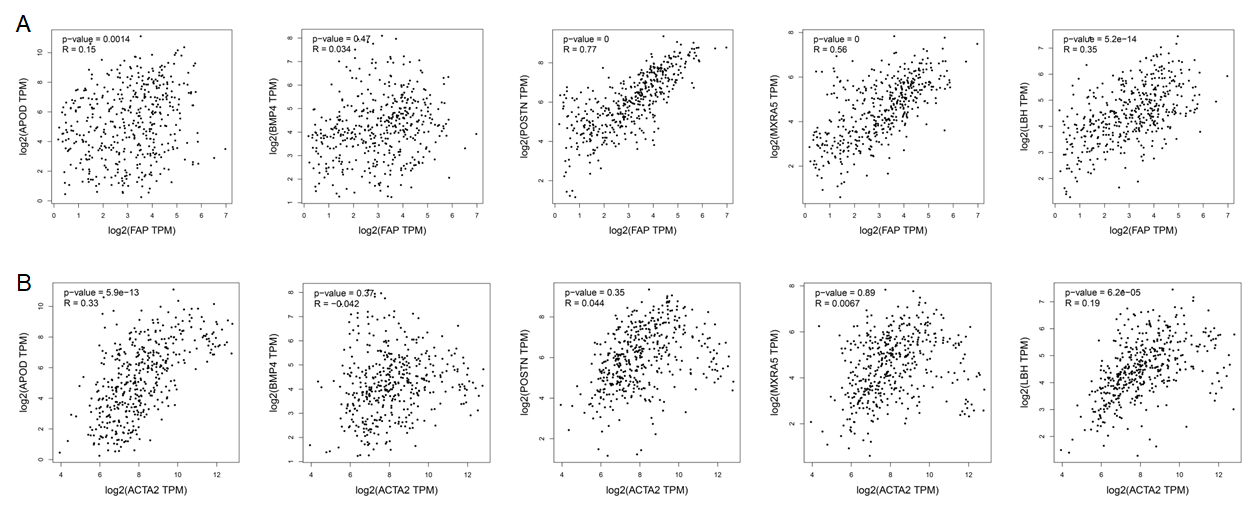


1. **Supplementary Figure S1.** A-B. The correlation between CAFs markers (ACTA2 and FAP) and hypoxia-related prognostic panel (including APOD, POSTN, BMP4, MXRA5 and LBH) were analysed using GEPIA2 database.


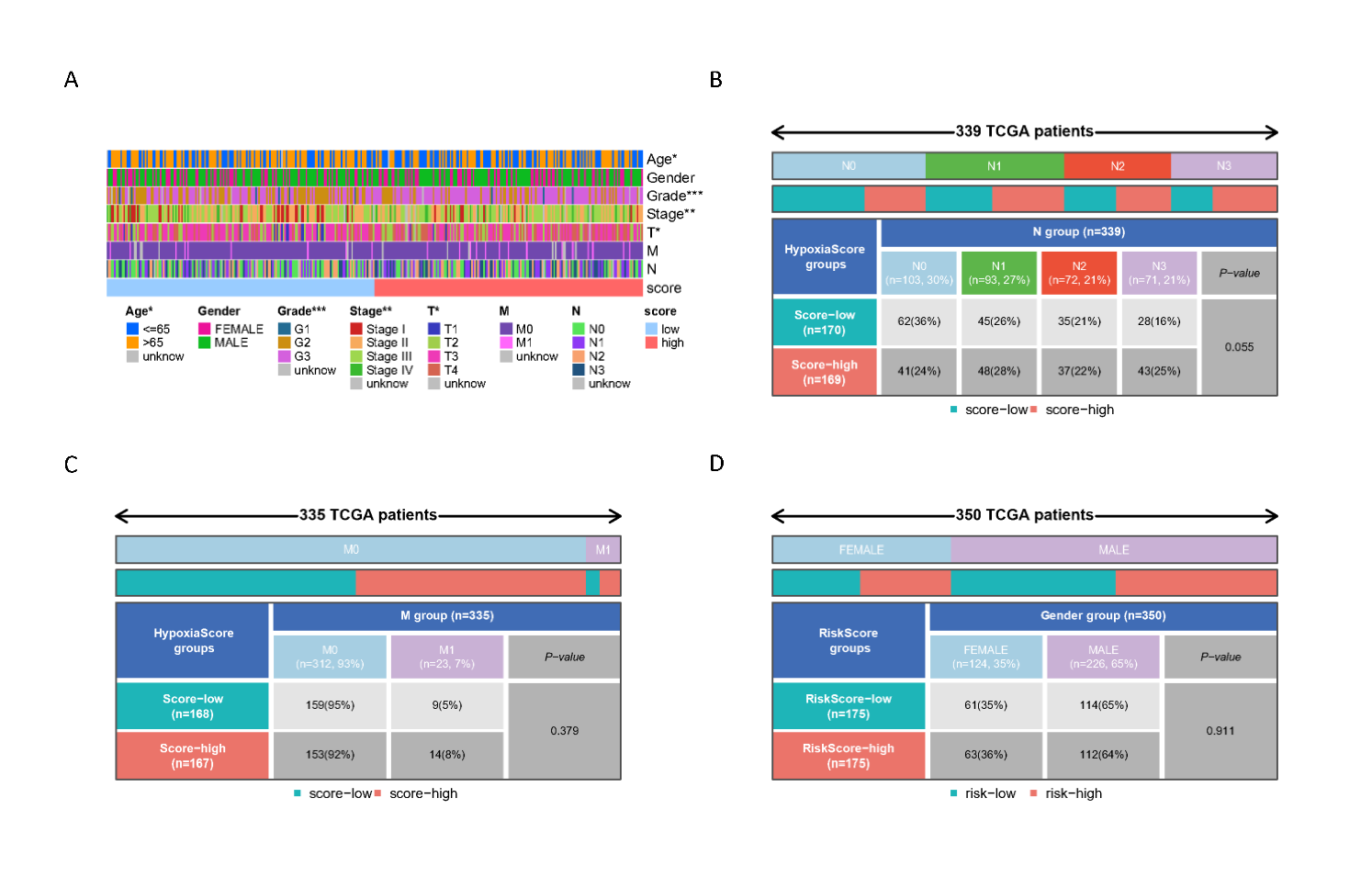


**Supplementary Figure S2.** A. Heatmap of the clinical characteristics of patients in different hypoxia score subgroups. B-D. There was no significant difference in sex, N stage or M stage between the two hypoxia scoring subgroups.


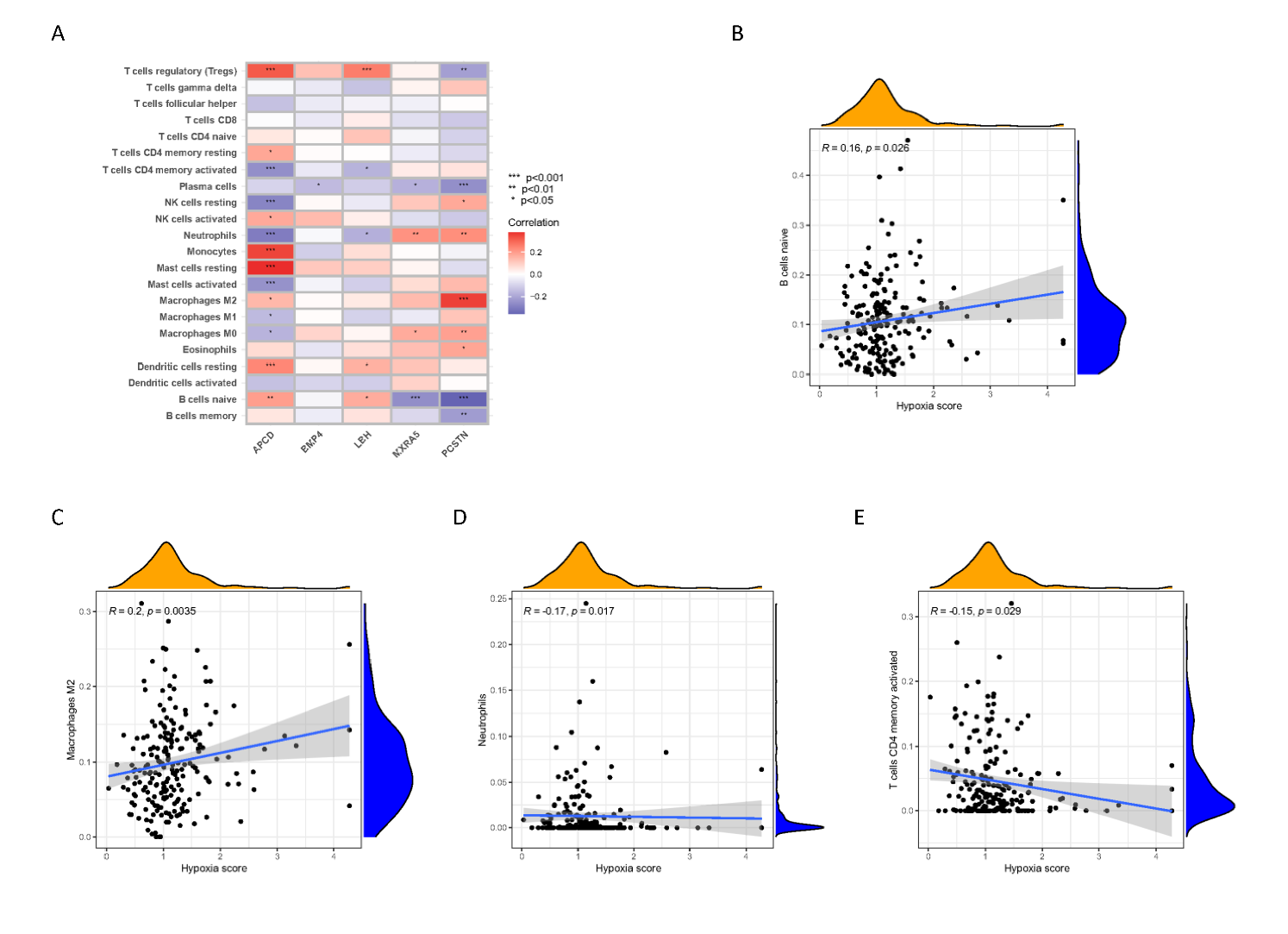


**Supplementary Figure S3.** A. The correlation analysis between the selected hypoxia-related genes and the immune cells according to the CIBERSORT. B-E. The correlation analysis between the hypoxia-score and B cells naïve, M2 macrophage, neutrophils and CD4 T cells based on CIBERSORT.


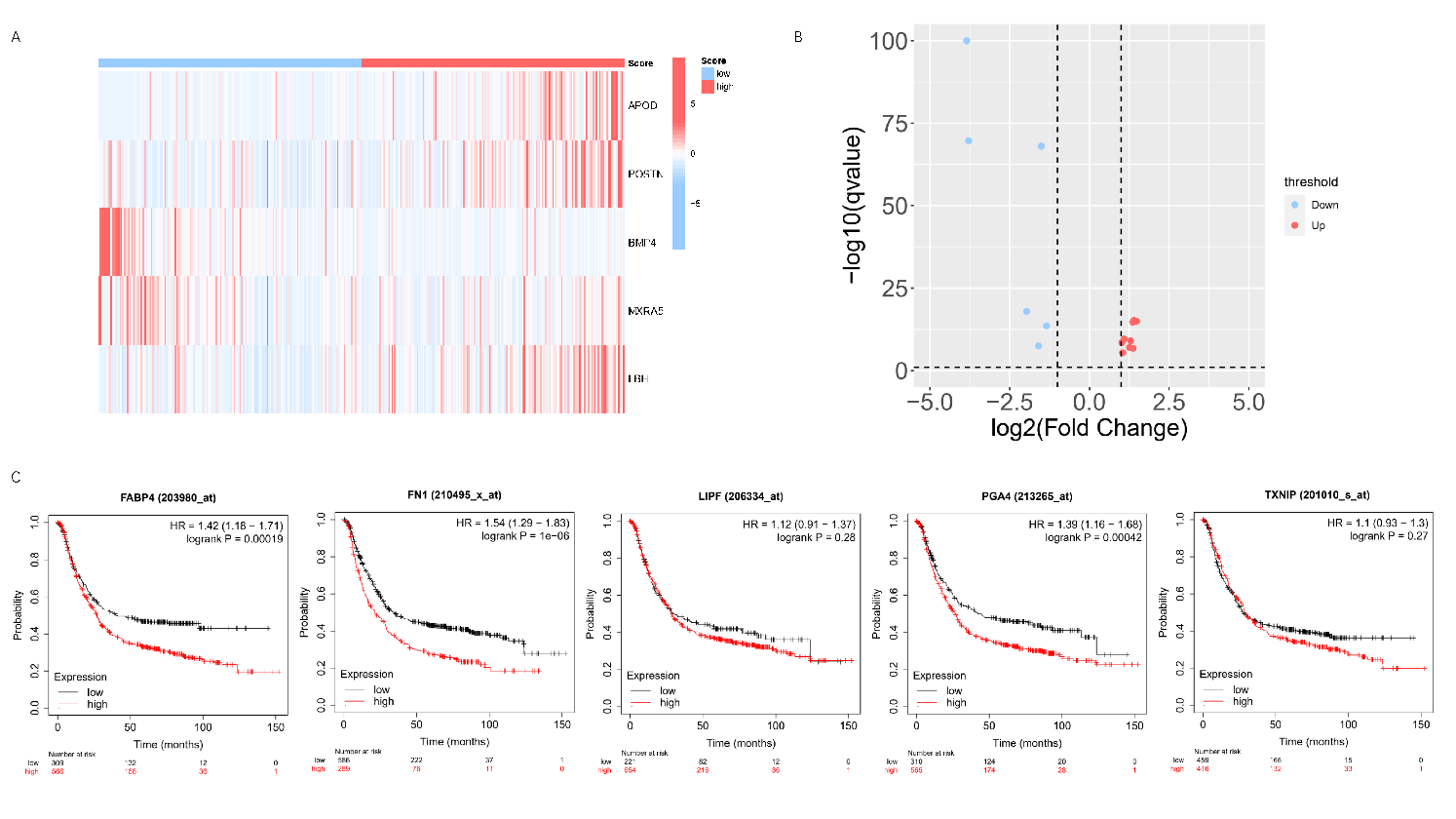


**Supplementary Figure S4.** A. Expression of hub hypoxia genes in different subgroups. B. Volcano of the different expressed genes in cluster 2 CAFs compared with other CAFs. C. Kaplan‒Meier analysis of selected hypoxia-related genes in the TCGA-GC cohort.

## Supplementary Tables

**Supplementary Table S1.** Primer sequences of hub genes for real-time polymerase chain reaction.

| Target | Sequence of primers |
| --- | --- |
| β-actin-Forward | CACCATTGGCAATGAGCGGTTC |
| β-actin-Reward | AGGTCTTTGCGGATGTCCACGT |
| BMP4-Forward | GCCAGCATGTCAGGATTAGC |
| BMP4-Reward | AATCCAGTCATTCCAGCCCA |
| LBH-Forward | GCCCCGACTATCTGAGATCG |
| LBH-Reward | GCGGTCAAAATCTGACGGGT |
| MXRA5-Forward | GCATCCCCGTGATTGTGATC |
| MXRA5-Reward | CATCTCTCTGTGTGGCATGC |
| POSTN-Forward | CCTTGGAAGAGACGGTCACT |
| POSTN-Reward | CTCAAAGACTGCTCCTCCCA |
| APOD-Forward | CTTTGAGAATGGACGCTGCA |
| APOD-Reward | TTCTCATAGTCGGTGGCCAG |

## Supplementary Table S2

Table S2. Differentially expressed genes in cluster 2 CAFs compared to other CAFs.

| Genes | *p*_val | log2FC | pct.1 | pct.2 | *p*_val_adj |
| --- | --- | --- | --- | --- | --- |
| PGA3 | 1.29E-121 | -5.87492832 | 0.029 | 0.627 | 3.42E-117 |
| LIPF | 4.08E-105 | -3.84535794 | 0.013 | 0.485 | 1.08E-100 |
| PGC | 9.15E-75 | -3.79015349 | 0.117 | 0.694 | 2.43E-70 |
| PGA5 | 3.79E-73 | -1.50748293 | 0.003 | 0.291 | 1.01E-68 |
| FABP4 | 4.31E-23 | -1.96668231 | 0.262 | 0.634 | 1.14E-18 |
| FN1 | 2.35E-20 | 1.397411728 | 0.747 | 0.321 | 6.23E-16 |
| COL1A1 | 4.33E-20 | 1.477162909 | 0.871 | 0.575 | 1.15E-15 |
| S100A4 | 7.38E-20 | 1.361472748 | 0.788 | 0.373 | 1.96E-15 |
| TXNIP | 1.08E-18 | -1.34377154 | 0.42 | 0.694 | 2.86E-14 |
| S100A6 | 1.05E-14 | 1.091346203 | 0.925 | 0.672 | 2.79E-10 |
| LOXL2 | 3.46E-14 | 1.296898175 | 0.682 | 0.366 | 9.21E-10 |
| MIR4435-2HG | 1.18E-13 | 1.019293749 | 0.76 | 0.44 | 3.14E-09 |
| IGFBP5 | 1.21E-12 | -1.59584109 | 0.227 | 0.478 | 3.21E-08 |
| TNC | 3.54E-12 | 1.257023591 | 0.384 | 0.067 | 9.41E-08 |
| S100A10 | 6.92E-12 | 1.370478557 | 0.548 | 0.239 | 1.84E-07 |
| EGFL6 | 1.46E-10 | 1.044559217 | 0.357 | 0.082 | 3.87E-06 |

Notes:1. Pct1(Percentage 1) refers to the proportion of cells expressing this gene compared to total cells in the tumor. 2. Pct2(percentage 2) refers to the proportion of cell expressing this gene in the normal compared to normal tissue.
